# Avoidant Attachment, Withdrawal-Aggression Conflict Pattern, and Relationship Satisfaction: A Mediational Dyadic Model

**DOI:** 10.3389/fpsyg.2021.794942

**Published:** 2022-01-31

**Authors:** Ione Bretaña, Itziar Alonso-Arbiol, Patricia Recio, Fernando Molero

**Affiliations:** ^1^Department of Clinical and Health Psychology and Research Methods, Faculty of Psychology, University of the Basque Country UPV/EHU, San Sebastian, Spain; ^2^Faculty of Psychology, Universidad Nacional de Educación a Distancia UNED, Madrid, Spain

**Keywords:** actor-partner interdependence model, conflict resolution, demand/aggression, mediation model, relationship satisfaction, romantic attachment, withdrawal

## Abstract

This study was conducted with the purpose of analyzing the combined and mediating effect of actor’s withdrawal–partner’s demand conflict resolution strategies between avoidance attachment dimension and relationship satisfaction. We conducted a dyadic study with 175 heterosexual couples (aged between 18 and 72 years) who filled in the questionnaires. Six hypotheses were tested using the actor–partner interdependence model with mediation analysis (APIMeM). Results showed that the avoidance dimension of attachment was more strongly associated with actor’s withdrawal strategy than with demand/aggression strategy. Furthermore, avoidance attachment was negatively associated with both actor’s and partner’s relationship satisfaction, the actor effect being higher. Withdrawal strategy was a mediator between actor’s avoidance and actor’s relationship satisfaction, but it was not a mediator for partner’s relationship satisfaction. The interactive pattern of actor’s withdrawal–partner’s demand/aggression was associated with low levels of both actor’s and partner’s relationship satisfaction. These results point out to the need of discerning the interactive pattern of conflict-solving strategies as well as their intertwined effect on relationship satisfaction.

## Introduction

Throughout history, close relationships have evolved. Many factors influence intimate relationships; some of them derive from current societal communication modes, such as social networks (Bridges and Morokoff, 2011; Howard, 2020), whereas some others cross boundaries of times and cultures, such as adult attachment (Abela et al., 2020). In fact, research in close relationships has identified insecure attachment as a powerful predictor of the reported low levels of relationship satisfaction (e.g., Li and Chan, 2012; Feeney, 2016). Moreover, several metaanalyses have shown that the avoidance attachment orientation accounts for a much stronger negative effect on relationship satisfaction as compared to anxiety attachment, this finding being documented in a wide range of situations and geographical locations (e.g., Li and Chan, 2012; Hadden et al., 2014; Candel and Turliuc, 2019). The specific mechanisms behind this association need to be ascertained, though, to gain knowledge for assisting couples in improving their relational experience.

A key mediating element may refer to how partners deal with their problems. In fact, inadequate conflict resolution predicts relationship satisfaction deterioration (Siffert and Schwarz, 2011) along with the appearance of more abusive and violent behaviors within the couple (Honeycutt et al., 2015), which could eventually lead to the chronification of dysfunctional relational patterns (e.g., Moral de la Rubia et al., 2011). Some conflict resolution strategies are better understood within such dysfunctional interactive conflict patterns (e.g., Christensen et al., 2007). A few researchers (e.g., Bonache et al., 2019; Bretaña et al., 2019, 2020) have recently observed a pattern in which the use of withdrawal conflict resolution strategy by one couple member (e.g., avoid the situation, not speak, and distance oneself) is linked with the use of demand/aggression strategy from her/his partner (e.g., criticize, demand, and threat). An individual’s conflict withdrawal reduces the likelihood of an adequate problem solving (Gottman, 1998), and his/her partner’s aggressive responses may increase due to the frustration generated by the situation (Miga et al., 2010). This may bring important negative consequences; for instance, Zamir and Lavee (2015) found that evasive emotion regulation strategies make it difficult to recognize abusive relationships.

A recent study (Bretaña et al., 2020) has provided preliminary evidence on the mediating effect of the aforementioned conflict resolution pattern between avoidance attachment orientation and relationship satisfaction from an individual perspective. Research about relational processes, though, requires a dyadic viewpoint for a more complete picture of this two-sided phenomenon (e.g., Brassard et al., 2009). Therefore, the present study was depicted with the specific aim of examining the mediating role of the withdraw–demand conflict pattern between avoidance attachment and relationship satisfaction in both the couple members. By embracing a dyadic perspective, disentangling further the interrelated dynamics of both partners’ conflict strategies became possible.

In the following sections, we will deepen into the theoretical underpinnings of the relational model of the conflict solving strategies used in the present study. This will be followed by an account of the empirical literature on avoidance attachment as related to conflict-solving strategies and relationship satisfaction.

### Avoidant Attachment, Conflict Resolution, and Relationship Satisfaction

Attachment theory is a useful theoretical framework to understand responses in an interactional process, such as the couple conflict (e.g., Mikulincer and Shaver, 2016) and changes in relationship satisfaction (Cooper et al., 2018). Differences in romantic attachment could be explained through two dimensions (Brennan et al., 1998): avoidance (of intimacy) and anxiety (about abandonment). While individuals scoring high in avoidance attachment may be described as those who need more independence and emotional distance from their partners to feel comfortable, individuals scoring high in anxiety attachment would be characterized by an excessive preoccupation and fear of being abandoned by their partner (Fournier et al., 2011). Despite their salience for relational dynamics, avoidant attachment is unmistakingly associated with lower scores of relationship satisfaction, as documented by several metaanalyses (e.g., Li and Chan, 2012; Hadden et al., 2014; Candel and Turliuc, 2019).

In this context, handling conflictive situations may be the interactive missing piece of the jigsaw puzzle. Conflictive situations bring to the activation of attachment system (Simpson et al., 1996; Lawler-Row et al., 2006). Such activation and its subsequent regulation exert an impact on the individual’s cognitive, emotional, and behavioral responses (Collins and Read, 1994; Zhang and Labouvie-Vief, 2004). Specifically, individual differences in attachment would account for the variability of their responses during the conflict (see Mikulincer and Shaver, 2016). Individuals scoring high on avoidant attachment tend to perceive conflict as a threat (e.g., Kobak and Duemmler, 1994), consequently deploying some inadequate resolution strategies (Pistole and Arricale, 2003; Shi, 2003).

Furthermore, it is not only that avoidant individuals’ biased interpretation makes them feel uncomfortable in situations of high intimacy, which leads to the avoidance behavior (Collins and Read, 1990),but also, they perceive that their partners are unable to adequately respond to their (avoidant people’s) needs, which in turn exerts a negative effect in their relationship satisfaction levels (Collins, 1996). Therefore, a partner’s perceived behaviors would be the response to one’s own behavior (Collins, 1996), the demand/aggression resolution-strategy being the consequence of one’s withdrawal of conflict. Avoidant individuals’ perception of a pressure to engage and getting close to their partner would lead them to using emotion regulation techniques of deactivation, which translates into avoiding the conflict to a higher extent, as shown in Bretaña et al. (2019, 2020) studies on perception of partners. Nevertheless, despite its demonstrated relevance in understanding conflict resolution and relationship satisfaction, the avoidant dimension of attachment has not received enough attention as a key variable, as claimed by Bretaña et al. (2020). Moreover, to the best of our knowledge, the dyadic analysis of both partners’ interrelated links between avoidance attachment orientation and negative conflict strategies has not been conducted so far.

Apart from avoidant attachment, relationship satisfaction may also be predicted by how couples deal with a conflict (Cummings and Davies, 2010). Relationship satisfaction also appeared to be affected by the perception of resolution strategies that an individual is using during conflicts. For instance, Bretaña et al. (2020) found that avoidance attachment dimension predicted one’s own withdrawal conflict resolution strategy, which, in turn, was associated with lower levels of relationship satisfaction. Furthermore, withdrawal strategy was observed to mediate between avoidant attachment dimension and relationship satisfaction. Nevertheless, these authors only analyzed actor effect. Examining the effect of an individual’s attachment on partner’s relationship satisfaction seems essential, since this has been previously reported to happen (e.g., Banse, 2004; Molero et al., 2011): the detrimental effect of (actor) avoidant attachment on partner’s relationship satisfaction was remarkably stronger (Molero et al., 2016). To cover this gap, our study wants to analyze how, in relation to avoidant attachment, actor conflict strategies exert an effect on partner’s conflict strategies and relationship satisfaction.

### Conflict Resolution Strategies as Mediators: Partner Effects

There are several studies showing that avoidant attachment is a predictor of relationship satisfaction at both actor and partner level (e.g., Brassard et al., 2009; Sierau and Herzberg, 2012). The literature on the prediction of both partners’ relationship satisfaction from withdrawal conflict resolution is scarcer, though. Overall et al. (2013) indicated that withdrawal is a strategy with a relatively low success to solve the discussion effectively, which would result in relationship deterioration (Woodin, 2011). From this viewpoint, withdrawal could be associated with a partner’s low relationship satisfaction. Sears et al. (2016) also found that marital disregard, as a behavior deployed during the marital conflict, predicted low scores on partner’s relationship satisfaction. Following this line, the behaviors characteristic of the withdrawal conflict resolution strategy could be understood as forms of directing contempt toward the partner, and stonewalling described by Gottman (1994) may be a clear example of it. Therefore, we expect that actor’s higher levels of withdrawal resolution strategy will be associated with lower levels of partner’s relationship satisfaction (Hypothesis 1).

Conflict-resolution strategies, specifically withdrawal, may be the missing puzzle piece to grasp the mechanisms underlying highly avoidant individuals’ (and their partners’) low relationship satisfaction. In fact, Brassard et al. (2009) found that conflict perception mediates between avoidant attachment and relationship satisfaction for both the actor and the partner. These authors analyzed how individuals perceive certain situations as more or less conflictive and their evaluation of such conflictive situations. Yet, they did not examine specific strategies (behaviors) used by individuals during the conflict. Knowing this aspect at a finer grain would allow us to understand which specific behaviors would lead to an inadequate conflict resolution and relationship satisfaction decline (e.g., Sanford, 2003). As a first step, when actor effects are examined, Sierau and Herzberg (2012) and Bretaña et al. (2020) have already provided evidence on the mediational role of withdrawal strategy between avoidant attachment and relationship satisfaction. From a dyadic perspective, and based on Cann et al.’s (2008) results regarding the mediating role of conflict resolution strategies between avoidance attachment and relationship satisfaction, Sierau and Herzberg (2012) suggested that avoidant individuals’ use of withdrawal strategy would predict partners’ low scores of relationship satisfaction. Consequently, and stemming from the evidence that attachment avoidance predicts conflict withdrawal (Sierau and Herzberg, 2012; Bretaña et al., 2020) and that avoidant attachment is a strong predictor of partner’s relationship satisfaction too (Molero et al., 2016; Candel and Turliuc, 2019), we hypothesize that withdrawal resolution strategy will mediate between actor’s avoidance attachment and partner’s relationship satisfaction (Hypothesis 2).

### Interrelations Between Maladaptive Conflict Solving Strategies and Relationship Satisfaction: Partner Effects

An unexplored but relevant question to the topic under study regards how highly avoidant individuals’ use of withdrawal conflict strategies is associated with the behavior displayed by their partners (i.e., demand strategy). Instances from the clinical work with couples point out to the relationship between actor’s withdrawal and partner’s demand strategy; specifically, Johnson (2004) observed that individuals’ withdrawal and/or silence during conflict (stonewalling), as response instances of conflict withdrawal, provoked their partners’ response of excessive criticism and demand/aggression. Indeed, conflict withdrawal is perceived by his/her partner as more harmful (Overall et al., 2013; Prager et al., 2019) and may cause him/her increased frustration (Johnson, 2004; Feeney and Karantzas, 2017). Consequently, his/her partner may react in a more aggressive way, which would lead both members to perceive the relationship as of a diminished quality. Eventually, this situation would bring both partners’ needs to be unmet, causing relational distress (Gottman, 1994).

In a series of studies, the demand/aggression strategy has been observed to be associated to lower relationship satisfaction for both actor (e.g., Eldridge et al., 2007) and partner (Bretz, 2009). Moreover, Segrin et al. (2009) and Sierau and Herzberg (2012) found that, it is not only the demand/aggression strategy, but rather all conflict-solving strategies that are not directed to a positive and effectively communicative resolution (i.e., withdrawal and demand/aggression) used by the individual what actually predicted both actor’s and partner’s lower relationship satisfaction. These investigations made an unquestionable contribution to the literature by analyzing conflict resolution strategies of both couple members simultaneously, and also their effect on relationship satisfaction; yet, the interrelations between actor’s strategy as related to partner’s were absent in the aforementioned studies. An in-depth understanding of negative interaction patterns (i.e., both partners deploying negative strategies) is relevant since those dynamics may lie behind an increase of the conflict (Crowley, 2008) and, therefore, of a progressive deterioration of the relationship.

In a study where interrelations of both partner’s identical negative conflict strategies (i.e., avoidant actor-avoidant partner and destructive actor-destructive partner) were examined for diminished relationship satisfaction (Bretz, 2009), associations between strategies were not found, though. Crowley (2008) argued that, in conflictive dynamics, partners tend to use opposite strategies in response to each other’s. Withdrawal and demand/aggression would be strategies more often used when coping abilities are lacking (Hurtado et al., 2004), which at the same time are susceptible to originate more negative dynamics (Christensen and Heavey, 1993). Bretaña et al. (2020) found that one’s own withdrawal strategy predicted the perception of partner’s higher use of demand/aggression strategy (which, in turn, predicted lower relationship satisfaction). However, while undoubtedly promising, these results relied on data of same individual’s perceptions; that is, a dyadic approach was not undertaken to test interrelations of strategies used by actor and partner. Therefore, in the present study, embracing a dyadic design, we expect that actor’s withdrawal conflict strategy will be associated with partner’s demand/aggression strategy (Hypothesis 3).

Regarding the interaction of dysfunctional strategies and the links with relationship satisfaction, Gottman (1994) observed that negative interactions (criticism, defensiveness, withdrawal, and contempt) exerted an unfavorable effect on the relationship quality. Likewise, Noller and Feeney (2002) also obtained evidence on the association of certain conflict strategy patterns and lower relationship satisfaction and dissolution. These results reveal the negative impact of negative and asymmetric conflict strategies on relationship quality. Thus, we expect that the interrelated pattern of maladaptive conflict solving strategies, where an actor withdraws and partner demands, —will be associated with actor’s lower levels of relationship satisfaction (Hypothesis 4). Furthermore, the links between actor’s withdrawal strategy and her/his partner’s demand/aggression strategy will be associated with partner’s lower levels of relationship satisfaction too (Hypothesis 5).

### Full Hypothesized Model (Hypothesis 6)

**FIGURE 1:**
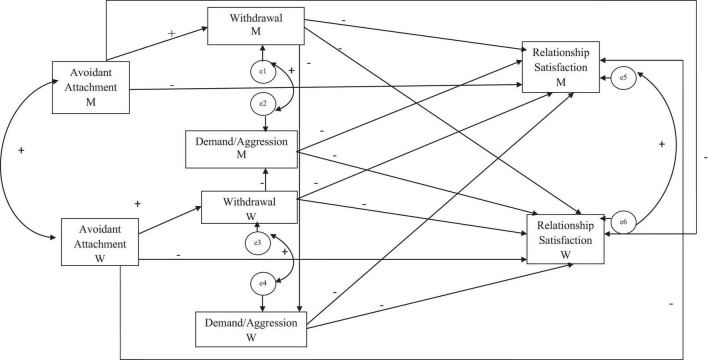
Hypothesized model.

Based upon the rationale explained above, a model of dyadic mediation is proposed here (Figure 1). In the overall model, we suggest that avoidant attachment dimension will be positively associated with own conflict resolution strategies, and these variables will be associated (actor and partner) with relationship satisfaction. Withdrawal conflict resolution strategy will mediate between avoidant attachment dimension and relationship satisfaction at both actor and partner levels. Finally, the interaction of conflict resolution strategies (i.e., actor’s withdrawal-partner’s demand/aggression) will negatively affect both the members’ (actor and partner) relationship satisfaction.

## Materials and Methods

### Participants and Procedure

The study sample was composed of 175 heterosexual couples (350 individuals).^1^ To determine the minimal sample size, a power analysis for linear multiple regression analysis was conducted. Assuming a small effect (*f* = 0.02), with alpha = 0.05, 95% power, and six predictors, 146 participants (couples in this case) would have been needed for the study (G*Power, Version 3.1.7, Faul et al., 2009). All participants completed the questionnaires individually. A snowball procedure was employed for data collection. Once ethical permission was granted from authors’ university, a wide array of popular cultural and leisure courses (non-formal education) was targeted for the study; these are annually offered to general population by the municipality of the city where the university is located. These courses are popular due to the wide offer and inexpensive nature (they have a low entry-fee, being free for unemployed people). Contacts through university colleagues and their networks were also targeted. After the aim of the study was explained, interested couples contacted the main author to arrange an appointment for data collection, which took place between January and April of 2019.

### Instruments

Participants filled in standardized self-reported questionnaires in paper-and-pencil format. The selection of standardized questionnaires is based on previous studies that have proved their validity and reliability with Spanish couples, the attachment questionnaire (Alonso-Arbiol et al., 2007, 2008), the relationship satisfaction questionnaire (Molero et al., 2011, 2016), and construct validity of all three measures (Bretaña et al., 2020). Furthermore, individuals completed a sheet with sociodemographic information with questions about age, marital status, relationship length, number of children, and education level. Demographic information of the sample can be seen in Table 1.

**TABLE 1:** Socio-demographic characteristics of the sample (*N* = 350 individuals).

Variables	*M* (*SD*)
Relationship length (range 0.5–47 years)	12.95 (10.24)
Age (range 18–72 years)	37.3 (10.17)
	Percentages
**Relationship status**	
Married	58.0%
Cohabiting	26.9%
Dating	15.1%
**Children**	
Yes	59.9%
No	40.1%
**Education level**	
Primary studies	6.8%
Secondary studies	32.9%
Higher education	60.3%
**Nationality/ethnicity**	
Spanish	98.3%
Latin-American (Colombian and Ecuadorian)	0.9%
Bangladeshi	0.6%
Dual nationality (United States and Spanish)	0.3%

***Experiences in close relationships*** (ECR) (Brennan et al., 1998; Spanish version by Alonso-Arbiol et al., 2007). This self-reported questionnaire taps the two dimensions of romantic attachment: anxiety (about relationships) and avoidance (of intimacy); however, in the current study we used only the avoidance dimension (18 items, to be rated on a Likert-7 scale, where 1 is “strongly disagree” and 7 “strongly agree”). One example of avoidance would be: “I prefer not to show my partner how I feel inside.” The total score was calculated averaging items’ score. High scores in this dimension represent higher levels of avoidance of intimacy. In our study, the internal consistency (Cronbach’s alpha) of this dimension was α = 0.84 (α = 0.80 for women and α = 0.86 for men).

***Conflict inventory revised*** (CI-R; Ridley et al., 2001; Spanish version by Bretaña et al., 2019). This inventory assesses responses during the couple conflict. The questionnaire has 13 items grouped into three subscales: positive problem solving (4 items), demand/aggression (4 items), and withdrawal (5 items). In the current study, we only analyzed the two maladaptive conflict resolution strategies: withdrawal (e.g., “hide tensions”) and demand/aggression (e.g., “blame my partner”). Items are rated using a Likert scale of 7 options, where 1 is “never” and 7 “always.” Higher scores represent higher use of those conflict resolution strategies. Cronbach’s alpha values were good for withdrawal subscale: α = 0.74 (α = 0.71 for women and α = 0.77 for men) and for demand/aggression subscale: α = 0.78 (α = 0.80 for women and α = 0.75 for men).

***Relationship assessment scale*** (RAS; Hendrick, 1988; Spanish version by Molero et al., 2016). This scale assesses the relationship satisfaction through seven items, for example: “My partner satisfied my necessities.” Items are rated using a Likert-7 scale, where 1 is “strongly disagree” and 7 “strongly agree.” Higher scores are indicative of higher relationship satisfaction levels. Internal consistency of the scale was good (α = 0.92 for total, and both women and men).

### Data Analyses

Differences between women and men were examined through mean comparison and Pearson correlation coefficients. A multivariate analysis of variance (MANOVA) was carried out to verify if there was a difference between women and men for the avoidance attachment dimension, conflict resolution strategies (withdrawal and demand/aggression), and relationship satisfaction. To test the hypotheses and to examine actor–partner effects, we used the APIM model (Kenny et al., 2006) with AMOS 23.0 (Arbuckle, 2014). The influence of individual’s independent variable on their dependent variable is called actor effect, while the effect of individual’s independent variable on their partner’s dependent variable is called partner effect. We analyzed the mediator role of conflict resolution strategies (i.e., withdrawal and demand) between avoidant attachment and relationship satisfaction using the bootstrap method (Cheung and Lau, 2008) separately with each mediator. The mediator effects were analyzed using a bootstrap procedure (5000 resamples) with 95% bias-corrected confidence interval. It is considered that, if zero is not included on the interval between the lower and the upper bound, the effect is statistically significant at p < 0.05. Nevertheless, this statistical significance is understood in a broader context of evaluating the effect size.

## Results

### Preliminary Analyses

Preliminary analyses show that only exist a statistically significant, yet small, association between age and relationship length with avoidant attachment dimension (*r* = 0.19, *p* < 0.001 and *r* = 0.17, *p* < 0.01, respectively).^2^ Age and relationship length showed no association with the remaining target variables of our study (i.e., own withdrawal, own demand/aggression, and marital satisfaction). The rest of sociodemographic variables analyzed (i.e., being parents and education level) did not show any association with our study variables (i.e., small effect size): avoidant attachment, actor withdrawal, actor demand/aggression, and marital satisfaction.^3^ Descriptive statistics and mean comparisons between men and women for target variables are presented in Table 2. Regarding MANOVA’s analysis, we found some small gender differences [*t*(248) = 2.06, *p* = 0.04, *d* = 0.22]; specifically, men (*M* = 2.44, *SD* = 0.89) showed higher scores of avoidance than women (*M* = 2.25, *SD* = 0.78), which is commonly the case in the literature.

**TABLE 2:** Descriptive data and gender differences for the target variables.

		Men	Women			
	Range	*M (SD)*	*M (SD)*	*t*	*p*	Cohen’s *d*
Avoidant attachment	1–7	2.44 (0.89)	2.25 (0.78)	2.06	0.04	0.22
Withdrawal	1–7	2.89 (1.10)	3.06 (1.10)	-1.52	0.78	0.02
Demand/aggression	1–7	1.56 (0.93)	1.77 (1.05)	-1.99	0.12	0.02
Relationship satisfaction	1–7	5.82 (1.30)	5.75 (1.35)	0.45	0.35	0.00

### Zero-Order Correlations Among Target Relational Variables

Correlations among variables of the study are displayed in Table 3 for men and women, separately. Avoidant attachment dimension correlated negatively with relationship satisfaction, showing a moderate effect size for both men and women. The correlation between avoidant attachment and withdrawal conflict resolution was positive and higher in both sexes than the correlation between avoidant attachment dimension and demand/aggression conflict resolution strategy.

**TABLE 3:** Correlations among target variables for men and women.

	1	2	3	4
1. Avoidant attachment	*0.84*	0.43**	0.39**	–0.58**
2. Withdrawal conflict strategy	0.50**	*0.74*	0.68**	–0.70**
3. Demand/aggression conflict strategy	0.39**	0.61**	*0.78*	–0.73**
4. Relationship satisfaction	–0.63**	–0.64**	–0.74**	*0.92*

*Correlations between women’s variables are displayed below the diagonal, while men’s correlations are displayed above the diagonal.*

*Cronbach alphas are shown in italics in the diagonal.*

**p < 0.01 (two-tailed).

Zero-order correlations between variables of the two couple members are displayed in Table 4. The correlation between actor’s avoidant attachment dimension and partner’s relationship satisfaction was negative and of moderate (similar) size for men and women (*r* = –0.52, *p* < 0.01 and *r* = –0.54, *p* < 0.01, respectively). Actor’s withdrawal conflict resolution strategy was positively and moderately associated with partner’s demand/aggression conflict resolution strategy (*r* = 0.54, *p* < 0.001 in the case of men’s withdrawal and women’s demand/aggression and *r* = 0.56, *p* < 0.001 for women’s withdrawal and men’s demand/aggression). Actor’s withdrawal and demand/aggression conflict resolution strategy were correlated negatively (and with a moderate-to-high size) with partner’s relationship satisfaction (*r* = −0.61, *p* < 0.001 and *r* = −0.70, *p* < 0.001 for men’s relationship satisfaction, respectively; and *r* = −0.62, *p* < 0.001 and *r* = −0.74, *p* < 0.001 for women’s relationship satisfaction, respectively).

**TABLE 4:** Dyadic correlations among study variables for coupled women and men.

	1 (M)	2 (M)	3 (M)	4 (M)
1. Avoidant attachment (W)	0.41**	0.28**	0.37**	–0.54**
2. Withdrawal conflict strategy (W)	0.24**	0.50**	0.56**	–0.61**
3. Demand/aggression conflict strategy (W)	0.37**	0.54**	0.74**	–0.70**
4. Relationship satisfaction (W)	–0.52**	–0.62**	–0.74**	0.89**

*W = Women and M = Men.*

*The diagonal, in boldface type, contains correlations with the same study variable between women and men.*

**p < 0.01 (two-tailed).

**FIGURE 2:**
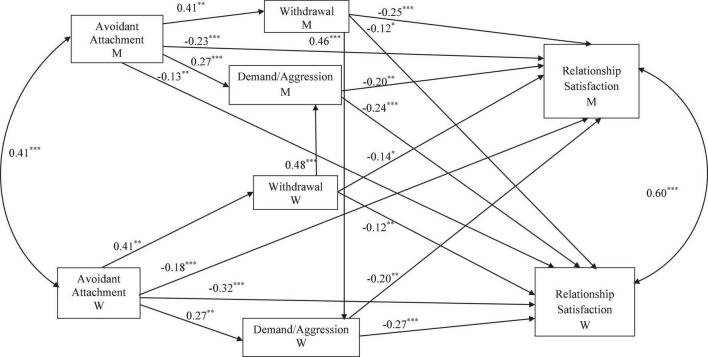
Actor-partner model for avoidant attachment dimension, withdrawal-demand conflict resolution strategies, and relationship satisfaction. M = Men, W = Women. Beta values are unstandardized. ^∗^*p* < 0.05; ^∗∗^*p* < 0.01; ^∗∗∗^*p* < 0.001.

The hypothesized model displayed in Figure 1 was tested (estimating all paths simultaneously). The initial model did not fit the data (χ*^2^*/*df* = 6.73, *p* < 0.01, AGFI = 0.69, CFI = 0.96, and RMSEA = 0.18). In the modification indexes subsection of the output, the model required to add a new path from avoidant attachment dimension to actor’s demand/aggression resolution strategy (Figure 2) to fit the data, which was eventually added due to its theoretical meaningfulness (for instance, Feeney and Karantzas, 2017). This improved model showed a good fit: χ*^2^/df* = 1.20, *p* = 0.30, AGFI = 0.94, CFI = 0.99, TLI = 0.99, RMSEA = 0.03.

### Actor Effects

As can been seen in Figure 2, both for men (β = 0.41, *p* < 0.001) and women (β = 0.41, *p* < 0.001), high scores in actor’s avoidant attachment dimension were positively associated with high scores in actor’s withdrawal conflict resolution strategy, to a greater extent than actor’s demand/aggression conflict resolution strategy (β = 0.27, *p* < 0.001 for men, and β = 0.27, *p* < 0.001 for women). Regarding demand/aggression conflict resolution strategy, it is associated with the actor’s relationship satisfaction. Nevertheless, although an actor’s demand/aggression explained low scores in the actor’s relationship satisfaction to a higher extent in women than in men (β = –0.27, *p* < 0.001 and β = –0.20, *p* < 0.01, respectively) when we compared the size of women’s and men’s actor effects, there was no significant gender differences [χ^2^Diff (1) = 0.22, *p* = 0.64].

Withdrawal conflict resolution strategy was positively associated with the actor’s relationship satisfaction. Regarding men’s actor effects, withdrawal was associated with low scores in actor’s relationship satisfaction to a higher extent in men than in women (β = –0.25, *p* < 0.001 and β = –0.12, *p* = 0.01, respectively). Nevertheless, when we compared the size of men’s and women’s actor effects, there was no significant gender differences [χ^2^Diff (1) = 2.10, *p* = 0.15].

### Partner Effects

Regarding Hypothesis H1, as can be observed in Figure 2, men’s withdrawal conflict strategy explained women’s low relationship satisfaction (β = –0.12, *p* = 0.02), and women’s withdrawal conflict strategy was also associated with men’s low relationship satisfaction (β = –0.14, *p* = 0.01). Thus, Hypothesis 1 was confirmed.

Indirect effects between avoidant attachment dimension and relationship satisfaction can be seen in Table 5. For both men and women, there was not a significant direct effect of avoidant attachment on relationship satisfaction, with all indirect paths set to zero. Therefore, withdrawal conflict resolution strategy did not mediate between actor’s avoidant attachment dimension and partner’s relationship satisfaction. Thus, Hypothesis 2 was not confirmed.

**TABLE 5:** Mediation effects in structural equation models.

Mediational analysis		Direct beta without mediation	Direct beta with mediation	Indirect beta [CI]
1. Avoidance Attachment (M)-Withdrawal (M)-Relationship Satisfaction (M)	Partial mediation	–0.58***	–0.35**	–0.22** [–0.31, –0.15]
2. Avoidance Attachment (W)-Withdrawal (W)-Relationship Satisfaction (W)	Partial mediation	–0.63***	–0.44***	–0.19** [–0.27, –0.12]
3. Avoidance Attachment (M)-Withdrawal (M)-Relationship Satisfaction (W)	No Mediation	–0.08	–0.01	–0.15** [–0.25, –0.07]
4. Avoidance Attachment (W)-Withdrawal (W)-Relationship Satisfaction (M)	No mediation	–0.02	0.04	–0.25** [–0.36, –0.14]
5. Avoidance Attachment (M)-Demand (M)-Relationship Satisfaction (M)	No mediation	–0.58***	–0.38***	–0.04 [–0.11, 0.04]
6. Avoidance Attachment (W)-Demand (W)-Relationship Satisfaction (W)	No mediation	–0.63***	–0.38***	–0.04 [–0.12, 0.03]
7. Avoidance Attachment (M)-Demand (M)-Relationship Satisfaction (W)	No mediation	–0.01	–0.00	–0.02 [–0.09, 0.05]
8. Avoidace Attachment (W)-Demand (W)-Relationship Satisfaction (M)	No mediation	–0.02	–0.01	–0.01 [–0.06, 0.02]
9. Withdrawal (M)-Demand (W)-Relationship Satisfaction (W)	Partial mediation	–0.43***	–0.26***	–0.31*** [–0.41, –0.19]
10. Withdrawal (W)-Demand (M)-Relationship Satisfaction (M)	Partial mediation	–0.61***	–0.42***	–0.23*** [–0.36, –0.09]
11. Withdrawal (M)-Demand (W)-Relationship Satisfaction (M)	Partial mediation	–0.59***	–0.32***	–0.29** [–0.41, –0.19]
12. Withdrawal (W)-Demand (M)-Relationship Satisfaction (W)	Partial mediation	–0.47***	–0.29***	–0.25*** [–0.35, –0.15]

*W = Women, M = Men.*

*Estimated values are standardized.*

**p < 0.01, ***p < 0.001.

Regarding the association between actor’s demand/aggression conflict resolution strategy and partner’s relationship satisfaction, in the case of men, actor’s demand/aggression strategy was negatively associated with the women’s (partner’s) relationship satisfaction (β = –0.24, *p* < 0.01); beta coefficient was of low-to-moderate size. Likewise, in the case of women, actor’s demand/aggression strategy also was negatively associated with men’s (partner’s) relationship satisfaction (β = –0.20, *p* < 0.01), the beta coefficient being of low-to-moderate size too.

As for the association between conflict resolution strategies interaction (partner effects), the results showed that actor’s withdrawal strategy was associated with partner’s demand/aggression strategy in men and women. Specifically, men’s withdrawal was positively associated with women’s demand/aggression (β = 0.46, *p* < 0.001), and women’s withdrawal was positively associated with men’s demand/aggression (β = 0.48, *p* < 0.001). In both cases, effect sizes were moderate. Hypothesis 3 was, therefore, confirmed.

### Direct and Indirect Effects of Actor’s Withdrawal and Relationship Satisfaction

Regarding Hypothesis 4, in men’s case, we observed an indirect effect between actor’s withdrawal and actor’s relationship satisfaction through partner’s demand/aggression (standardized indirect effect = –0.29, *SE* = 0.05, *p* < 0.01), which was statistically significant at the 95% confidence interval (95% CI = –0.41 to –0.19). Regarding women, we observed an indirect effect between the actor’s withdrawal and the actor’s relationship satisfaction through partner’s demand/aggression (standardized indirect effect = –0.25, *SE* = 0.05, *p* < 0.01), which was statistically significant at the 95% confidence interval (95% CI = –0.35 to –0.15). Therefore, Hypothesis 4 was confirmed.

Regarding the relationship between women’s withdrawal and men’s relationship satisfaction through men’s demand/aggression, we observed an indirect effect (standardized indirect effect = –0.23, *SE* = 0.07, *p* < 0.01), which was statistically significant at the 95% confidence interval (95% CI = –0.36 to –0.09). Regarding the relationship between men’s withdrawal and women’s relationship satisfaction through women’s demand/aggression, we observed an indirect effect (standardized indirect effect = –0.31, *SE* = 0.05, *p* < 0.01), which was statistically significant at the 95% confidence interval (95% CI = –0.41 to –0.19).^2^ Thus, Hypothesis 5 was confirmed.

To sum up, the overall model (Hypothesis 6) that explains the relationships among actor’s avoidant attachment, interactive (actor’s) withdrawal and (partner’s) demand/aggression conflict resolution strategies, and the relationships’ satisfaction (actor and partner) fit in as expected.

## Discussion

The main result of our study was the positive association between conflict withdrawal (actor) and demand conflict strategy (partner). This result would confirm what other authors (Bonache et al., 2019; Bretaña et al., 2020) found just at an individual level, whereas our data would extend the observation to a dyadic level. Thus, such results show that withdrawal deployed by one individual may elicit a negative emotional reaction in his/her partner (Miga et al., 2010; Feeney and Karantzas, 2017), which, in turn, would explain the demand/aggression response since withdrawal is understood as a defensive strategy of depreciative nature (Creasey and Ladd, 2004).

Avoidant attachment dimension is a clear predictor of relationship satisfaction. In our study, we found that avoidant attachment dimension predicts low scores in relationship satisfaction, at both the actor and partner level. Other research studies had also found similar results (Banse, 2004; Molero et al., 2011). Nevertheless, actor effects in our study were more visible than partner effects, something that had been highlighted by other authors (Molero et al., 2011; Orth, 2013). Thus, it seems that avoidant attachment, defined by discomfort with excessive closeness to partner, would increase the chances of becoming unhappy within the close relationship but would affect less partner’s perception of satisfactory marital life (Li and Chan, 2012). Jackson and Kirkpatrick (2007) argued that avoidance dimension was specifically associated with a lower inclination to be involved in long-term relationship due to their inner motivation to avoid intimacy and perceive close relationships as less gratifying, which led them fly when affective comfort ability diminishes. An indication of this could be grasped in stronger links of avoidance dimension of attachment, as compared to anxiety, with relationship dissatisfaction (Brassard et al., 2009; Molero et al., 2017). Avoidantly attached individuals’ partners, instead, may not perceive the relationship quality as worsened because they have become acquainted with their relational dynamics; thus, avoidant individuals’ relationships are shorter because they themselves put an end to it (Jang et al., 2002).

The main objective of our study revolved around the idea of unfolding the mediating value of the withdrawal conflict resolution strategy between avoidant attachment and relationship satisfaction. Our results corroborated this effect only at actor level, though. In fact, although avoidant attachment dimension and withdrawal conflict resolution strategy predicted partner’s relationship satisfaction, our study showed that actor withdrawal did not mediate between actor avoidant attachment and partner relationship satisfaction. The inexistence of mediation is not completely surprising due to the relatively low association between actor attachment avoidance and partner relationship satisfaction. This finding is in line with Sierau and Herzberg’s (2012) results; although they observed an indirect effect between avoidant attachment and partner’s relationship satisfaction, in the whole model, the relationship between withdrawal strategy and partner’s relationship satisfaction was not found. Following these authors’ argumentation line, avoidantly attached partners may activate a compensatory mechanism that would diminish the impact of withdrawal behaviors on these individuals’ assessment of their relationship. In samples composed by couples in long-lasting relationships, as it was the case of Sierau and Herzberg’s (2012) as well as in our own study, individuals (partners) could respond by using some strategies that compensate the negative effects of (actors) withdrawal on partners’ relationship satisfaction. Such compensatory mechanisms would be instrumental in helping them to counteract those negative aspects by coloring the relationship with positive elements, and therefore, making it more beneficial to remain in the relationship than terminating it (for a review, see Song et al., 2019). This is a fertile area for future exploration. Forthcoming research may benefit from conducting qualitative studies that further deepens into self-reported subjective meanings of partners’ responses and their connections with both members’ relationship satisfaction accounts. Accurate perceptions about specific partner responses have been associated with some positive behaviors (e.g., supporting behaviors) and negative behaviors (e.g., control of marriage and divorce), as Neff and Karney (2005) pointed out.

Finally, the relevance of demand/aggression in the model is noteworthy. The avoidant attachment dimension most often has appeared in the literature as being associated with the withdrawal conflict resolution strategy; commonly these individuals would use it as a preferent strategy to emotionally regulate themselves. Nevertheless, in the current study, we have found that avoidance attachment was also associated, to a lower extent, though with the demand/aggression strategy. Although this may seem contradictory at a first glance, this result is in consonance with other research studies’ results (Sierau and Herzberg, 2012; Martin et al., 2019). As Sierau and Herzberg (2012) suggested, depending on the situation, for instance, when escaping from the conflict seems more complicated (Feeney and Karantzas, 2017), avoidantly attached individuals may change their conflict resolution strategy; specifically, they may switch to a demand/aggression strategy as a way to cope with the uncomfortable relational situation. The use of this alternative strategy could answer to these individuals’ necessity to finish the discussion earlier (Sierau and Herzberg, 2012) or to prevent the excessive closeness from the partner (Mayseless, 1991). It may be of interest to analyze this strategy change in consonance with the nature of the situation, especially relevant nowadays when most countries have experienced a quarantine scenario having all inhabitants locked down and without any possibility of physically escaping from the couple conflict due to the COVID-19 pandemic.

## Conclusion

The aim of the current study was twofold: on the one hand, we aimed at analyzing the mediating effect of actor withdrawal conflict resolution strategy between actor avoidance attachment and actor–partner relationship satisfaction. On the other hand, we wanted to examine the withdrawal–demand/aggression pattern and its effect on relationship satisfaction. In both cases, the model was analyzed from a dyadic perspective. The main results showed, first, that withdrawal strategy would have a mediating effect between avoidant attachment dimension and relationship satisfaction (actor level). Additionally, an interesting new finding that highlights the role of conflict withdrawal was the existence of the links between actor withdrawal and partner demand/aggression, as well as the negative effect of this pattern in both own and partner relationship satisfaction. These results are enlightening within the framework of attachment theory to understand how individuals’ needs elicit specific responses in their partners. The interaction of both partners’ responses in stressful situations, which would reflect the quality of the affective bond and of the mental models that are activated, will be the key factors that help to understand both the course of the conflict and couple satisfaction.

In summary, our results help understanding avoidantly attached individuals’ conflict cycle. Nevertheless, certain limitations must be acknowledged. Firstly, the study was carried out with a community sample of Spanish heterosexual couples of a medium-high socioeconomic status. It could be advisable to conduct further research by including individuals of lower education levels and from other countries for the generalizability of results to the general population. Secondly, the present study is of crosssectional nature; thus, longitudinal designs may be adopted. In this regard, withdrawal has been longitudinally associated with lower relationship satisfaction (Christensen and Heavey, 1990; Heavey et al., 1995; Woodin, 2011), and withdrawal behaviors seem to be better predictors of relationship satisfaction in distressed couples than in non-distress ones (Mondor et al., 2011). Therefore, future research could incorporate several measure times of relationship satisfaction to reveal causality in dyadic dynamics derived from conflict resolution strategies and the possible negative progression of those strategies on relationship satisfaction controlling for the initial relationship distress levels. Furthermore, the newest communication modes through technology are ubiquitous (McDaniel and Drouin, 2015), which has changed the forms and easiness of avoiding the conflict. Therefore, these new scenarios for conflict resolution need to be incorporated in future studies. In fact, avoidantly attached individuals show higher levels of relationship satisfaction sending erotic contents through the mobile phone (i.e., sexting) in a more secure and a less conflictive environment (McDaniel and Drouin, 2015). Finally, conflict resolution strategies were only assessed with self-report questionnaires. Interview procedures may be used in the future to clarify the functions of each strategy and behaviors deployed during the conflict or a combination of observation and self-questionnaire assessment methods. While there has been a long tradition of using observational measures to assess conflict resolution, we reckon that some behavioral characteristics of withdrawal-demand/aggression, or of the reversed pattern (e.g., escape from the physical scenario), could not be observed in a laboratory situation, as Caughlin and Reznik (2016) pointed out. In fact, in the metaanalysis by Schrodt et al. (2014), the combined use of self-questionnaires and behavioral procedures appeared as a powerful moderator between demand/aggression–withdrawal pattern and relational, communication, or wellbeing variables, whereas the effect of using either observation or surveys was similar.

In a nutshell, our study sheds light to understand the interactive nature of the conflict strategies used by avoidantly attached individuals and how those are linked to relationship outcomes. In more practical terms, it is necessary to know the associations between withdrawal and demand/aggression to better discern some aspects linked with victimization and abusive behaviors that avoidantly attached individuals may be involved in Bonache et al. (2019). Focusing on the appearance of the withdrawal-demand/aggression maladaptive pattern would be instrumental for couple therapists as it brings couples’ general inadequate functioning (Shoham and Rohrbaugh, 2002) and adds discomfort derived from the perpetuation of cycles of negative conflict strategies (Papp et al., 2009). We believe that our findings may be useful for those professionals working with avoidant individuals in close relationships, so that they can provide them with tools to cope with conflict and detect negative or maladaptive conflict processes (Siffert and Schwarz, 2011).

## Data Availability Statement

The raw data supporting the conclusions of this article will be made available by the authors, without undue reservation.

## Ethics Statement

The studies involving human participants were reviewed and approved by the Ethics Committee for Research Involving Human Beings (CEISH) of the University of the Basque Country UPV/EHU (M10/2016/131). The patients/participants provided their written informed consent to participate in this study.

## Author Contributions

IB and IA-A planned the study and drafted the manuscript. IB, IA-A, and FM coordinated the data collection. IB, IA-A, and PR designed and performed the calculations. All authors discussed the results, and contributed to all versions of the manuscript.

## Conflict of Interest

The authors declare that the research was conducted in the absence of any commercial or financial relationships that could be construed as a potential conflict of interest.

## Publisher’s Note

All claims expressed in this article are solely those of the authors and do not necessarily represent those of their affiliated organizations, or those of the publisher, the editors and the reviewers. Any product that may be evaluated in this article, or claim that may be made by its manufacturer, is not guaranteed or endorsed by the publisher.

## Footnotes

1 According to data provided by the Spanish National Institute of Statistics (INE, 2021), 83.8% of couples are married, while 16.2% are cohabiting. Regarding parenting, more than half of sample of our study informs of having children. According to data of Spanish population, 38.7% of Spanish couples are not parents, whereas 61.3% are parents. Therefore, we may conclude that our study does not differ much from the general population in these sociodemographic variables. However, regarding education level, our study sample is more educated than the average population.

2 We found gender differences in the correlation between age and avoidant attachment, on the one hand, and between relationship length and avoidant attachment, on the other hand. The correlation between men’s age and avoidant attachment dimension was low (*r* = 0.12, *p* = 0.11). The correlation between women’s age and avoidant attachment dimension was statistically significant but was low-to-moderate (*r* = 0.26, *p* < 0.001). Regarding relationship length, it did not correlate with avoidant attachment for men (*r* = 0.12, *p* = 0.11), while the correlation for women was low-to-moderate (*r* = 0.22, *p* = 0.00). As some authors found in their metaanalysis study (e.g., Candel and Turliuc, 2019), individual’s age and relationship length mediated between avoidant attachment dimension and relationship satisfaction (actor level); therefore, in the current study we decided to control for these variables within our model. The results showed that the model including these control variables did not fit after controlling for them (χ^2^/*df* = 27.94, *p* = 0.00, CFI = 0.62, TLI = 0.09, RMSEA = 0.39).

3 Regarding sociodemographic variables (i.e., age, gender, education level, being a parent, relationship length, and time living together), after analyzing each one separately, none showed a statistically significant relationship with relationship satisfaction. The beta values for the regression weight of these variables predicting relationship satisfaction were as follows: age (β = –0.10, *t* = –0.98, *p* = 0.33); gender (β = –0.01, *t* = –0.18, *p* = 0.85); education level (β = 0.00, *t* = 0.00, *p* = 0.99); being a parent (β = 0.06, *t* = 1.15, *p* = 0.25); relationship length (β = 0.07, *t* = 0.47, *p* = 0.63); and time living together (β = –0.00, *t* = –0.03, *p* = 0.97).
